# Autoimmune nodopathy associated with contactin-2 antibodies manifesting as Guillain–Barré syndrome: a case report

**DOI:** 10.1186/s13256-026-06195-5

**Published:** 2026-06-05

**Authors:** Xiaoxia Zeng, Juanjuan Hu, Ke Li, Zhiji Gan, Xueliang Qi

**Affiliations:** Department of Neurology, The Second Affiliated Hospital of Nanchang University, Nanchang, 330006 Jiangxi Province China

**Keywords:** CNTN2, Autoimmune nodopathy, Clinical manifestation characteristics, EMG, Treatment, Case report

## Abstract

**Background:**

The autoimmune nodopathy affecting the node of Ranvier was formerly classified within the spectrum of chronic inflammatory demyelinating polyradiculoneuropathy. However, as a result of comprehensive pathological and immunological investigations conducted in recent years, it has increasingly been recognized as a distinct clinical entity. To date, there have been no reported cases linking autoimmune nodopathy at the node of Ranvier with anti-CNTN2 antibodies.

This paper presents a case study demonstrating such an association, detailing the clinical and electrophysiological features, and thereby contributing to the global understanding and recognition of this condition.

**Case presentation:**

The patient, a 48-year-old female of Han nationality, was admitted to the intensive care unit (ICU) after experiencing a period of unconsciousness, accompanied by a high-grade fever lasting over 4 h. Diagnostic evaluations, including blood tests and imaging studies, indicated the presence of heat stroke and coagulation dysfunction. The therapeutic interventions administered included endotracheal intubation and mechanical ventilation, continuous cooling with electric ice blankets and ice caps, treatments to correct coagulopathy, and aggressive fluid resuscitation. Following these interventions, the patient regained consciousness, and her body temperature returned to normal. However, upon cessation of mechanical ventilation, she exhibited limb weakness and produced indistinct vocalizations, although she was capable of sound production. Routine electromyography identified peripheral nerve injury of the axonal type, while cerebrospinal fluid analysis revealed protein-cell dissociation. Laboratory assays of both blood and cerebrospinal fluid samples tested positive for the anti-CNTN2 antibody IgG. Given the limited availability of effective therapeutic research for this condition at the time, intravenous immunoglobulin therapy was administered with the patient's informed consent, although it did not result in significant improvement of her symptoms.

**Conclusion:**

Clinically, impairments in limb motor abilities, dysarthria, respiratory insufficiency, protein-cell dissociation within the cerebrospinal fluid, and early axonal degeneration as evidenced by electromyography are critical diagnostic criteria for autoimmune nodopathy of the Ranvier nodes in patients who test seropositive for anti-CNTN2 antibodies. For individuals presenting with these phenotypes and suspected of having Guillain-Barré syndrome or chronic inflammatory demyelinating polyradiculoneuropathy, it is essential to conduct comprehensive assessments for node, paranode, and juxtaparanode antibodies, along with their specific subtypes, to refine therapeutic strategies.

## Introduction

Autoimmune nodopathy (AN) is a group of immune-mediated peripheral neuropathy, clinical manifestations of multiple motor-sensory peripheral neuropathy, AN and chronic inflammatory demyelinating polyradiculoneuropathy (CIDP) have a similar clinical presentation, subtypes previously classified in CIDP. In recent years, advancing research has led to the classification of autoimmune nodopathy (AN) as a distinct disease entity by the European Academy of Neurology (EAN) and the Peripheral Nerve Society (PNS) in 2021. The node of Ranvier is a critical structure facilitating saltatory conduction in myelinated peripheral nerve fibers, comprising nodal, paranodal, and juxtaparanodal regions, each characterized by distinct molecular compositions [1]. An imbalance in immune function can result in the production of antibodies targeting these molecular structures, leading to peripheral neuropathy. Antibodies against the node of Ranvier can be identified in the serum of AN patients, including those with peripheral neuropathy associated with neurofascin 155, contactin protein 1, and contactin-associated protein-1 antibodies. Contactin-2 (CNTN2) is another protein located in the juxtaparanodal regions [2]. To date, there have been no reported cases of AN associated with CNTN2 antibodies. Herein, we present what is likely the first documented case of autoimmune nodopathy associated with CNTN2 antibodies, detailing its clinical and electrophysiological characteristics.

## Case report

A 48-year-old Han Chinese woman was admitted to our hospital on August 20, 2022, where she presented with unconsciousness and a high fever that had persisted for over 1 day. One day prior to admission, she fainted after a 40-min walk in hot weather and subsequently became unresponsive. She did not experience any episodes of nausea, vomiting, or convulsions. Upon her admission to a local hospital, her temperature was recorded at 41.2 °C, and she was in a comatose state, exhibiting bradypnea and labored breathing. The local hospital diagnosed her with heat stroke and initiated treatment that included physical cooling, tracheostomy, and mechanical ventilation, which resulted in a temperature reduction to 38 °C. On August 20, 2022, she was subsequently transferred to our hospital. The patient was in good health before the onset of disease.

The physical examination revealed a temperature of 37.4 °C, a pulse of 77 beats per minute, mechanical ventilation with a respiratory rate of 26 breaths per minute, and a blood pressure of 104/84 mmHg (1 mmHg = 0.133 kPa). The patient was unconscious, exhibited diffuse ecchymosis, and had equal, round pupils measuring approximately 2 mm in diameter, with no light reflex. Auscultation revealed decreased breath sounds bilaterally, without significant dry or wet rales. The cardiac examination revealed a regular rhythm without notable murmurs, and the abdomen was soft with no palpable liver or spleen. The assessment of muscle strength and tone in the limbs revealed that the limb was uncooperative, and no pathological signs were elicited in either lower limb.

Laboratory examinations revealed a platelet count of 9 × 10^9^/L [(125–350) × 10^9^/L], a white blood cell count of 18.06 × 10^9^/L, a red blood cell count of 4.09 × 10^12^/L, a prothrombin time of 20.90 s (normal range: 9–13 s), a fibrinogen level of 1.63 g/L (normal range: 2–4 g/L), a creatine kinase level of 1681.60 U/L (normal range: 40–200 U/L), and a myoglobin level of 1336.50 μg/L (normal range: 0–100 μg/L). Chest CT showed a small amount of inflammation in both lungs. A CT of the head, abdomen, and pelvis revealed no significant abnormalities. The clinical diagnoses included heat stroke and coagulopathy. The patient continued to receive tracheostomy care, mechanical ventilation, physical cooling through the use of an ice blanket and cap, and rapid fluid resuscitation. From August 20 to August 21, 2022, she underwent platelet transfusions, virus-inactivated frozen plasma, coagulation factors, and leukocyte-free suspended red blood cells.

**Fig. 1:**
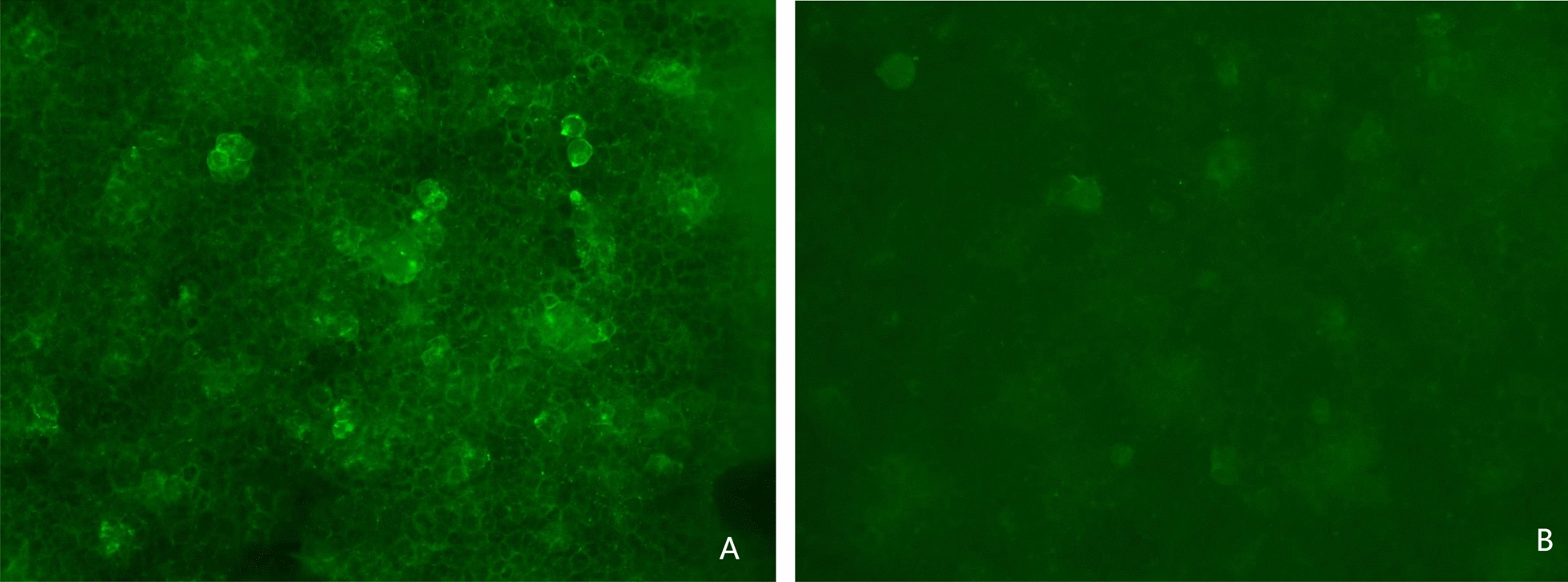
**A** the test of contactin-2 antibody in the serum was positive. **B** the test of contactin-2 antibody in cerebrospinal fluid was positive

Two days later, on August 22, the patient's consciousness returned, her body temperature normalized, and her condition gradually improved. Following the removal of the ventilator, the patient was able to produce sounds but could not articulate clearly. The examination of other cranial nerves yielded unremarkable findings. Muscle strength in the proximal extremities was Grade 2, whereas that in the distal extremities was Grade 0. Ataxia examination was uncooperative; however, sensory function remained intact, tendon reflexes were absent, and bilateral pathological signs as well as meningeal irritation signs were negative. Lumbar puncture was conducted on September 5, 2022. The cerebrospinal fluid pressure was measured at 125 mmH_2_O (1 mmH_2_O = 9.81 × 10^–3^ kPa; normal range: 80–180 mm H_2_O), with a white blood cell count of 10 × 10^6^/L [normal range: (0–10) × 10^6^/L] and a protein concentration of 1250 mg/L (normal range: 150–450 mg/L). Glucose and chloride levels were within the normal range. Antibodies against the ganglioside CNTN2-IgG were detected as positive in both cerebrospinal fluid and serum (CSF: 1:1; serum: 1:10) (Fig. 1), whereas antibodies against NF155, NF186, CNTN1, and Caspr1 were negative. Autoantibodies for GBS(AMAN variant) were negative. Electromyography (EMG) revealed a decrease in the amplitude of the compound action potentials in the peripheral nerves, whereas the amplitude and conduction velocity of the sensory potentials remained normal(Table 1). Intravenous immunoglobulin (IVIg) was administered at a dosage of 0.40 g/(kg·day) for five consecutive days in conjunction with bedside rehabilitation training. The patient exhibited improvement in distal weakness of the upper extremities and was subsequently transferred 33 days after hospitalization on September 22, 2022.

**Table 1:** The electromyography study of the patient

Site	Normal range	Left	Right
Distal motor latency (ms)			
Median: Wrist-APB	≤ 3.70	–	3.60
Ulnar: Wrist-ADM	≤ 2.90	–	3.20 (extended by 0.30 ms, an extension of 10.34%)
Peroneal: Ankle-EDB	≤ 5.00	–	–
Tibial: Ankle-AHB	≤ 4.80	3.60	4.70
Motor amplitude (mV)			
Median: Elbow-Wrist	≥ 8.00	–	0.70 (decreased by 7.30, a decrease of 91.25%)
Ulnar: Elbow-Wrist	≥ 8.00	–	0.80 (decreased by 7.20, a decrease of 90.00%)
Peroneal: Head-Ankle	≥ 3.00	–	–
Tibial: Ankle-AHM	≥ 6.00	1.30(decreased by 4.70, a decrease of 78.33%)	1.20 (decreased by 4.80, a decrease of 80.00%)
Motor conduction velocity (m/s)			
Median: Elbow-Wrist	≥ 56.00	–	57.90
Ulnar: Elbow-Wrist	≥ 61.00	–	61.50
Peroneal: Head-Ankle	≥ 44.00	–	–
Sensory amplitude (uV)			
Median: DigIII-Wrist	≥ 7.00	–	12.50
Ulnar: DigV-Wrist	≥ 7.00	–	11.70
Peroneal	≥ 8.00	12.70	11.40
Sural	≥ 0.90	10.30	8.80
Sensory conduction velocity (m/s)			
Median: DigIII-Wrist	≥ 55.00	–	52.10 (decreased by 2.90, a decrease of 5.27%)
Ulnar: DigV-Wrist	≥ 48.00	–	53.30
Peroneal	≥ 40.00	63.20	57.60
Sural	≥ 35.10	59.20	58.10
F-responses (%)			
Median: Wrist-APB	≥ 50.00	–	–
Ulnar: Wrist-ADM	≥ 50.00	–	–
Tibial: Ankle-AHB	≥ 50.00	–	–

ADM: Abductor Digiti Minimi; EDB: Extensor Digitorum Brevis; AHB: Abductor Hallucis Brevis; AHM: Abductor Hallucis Minimi

**Fig. 2:**
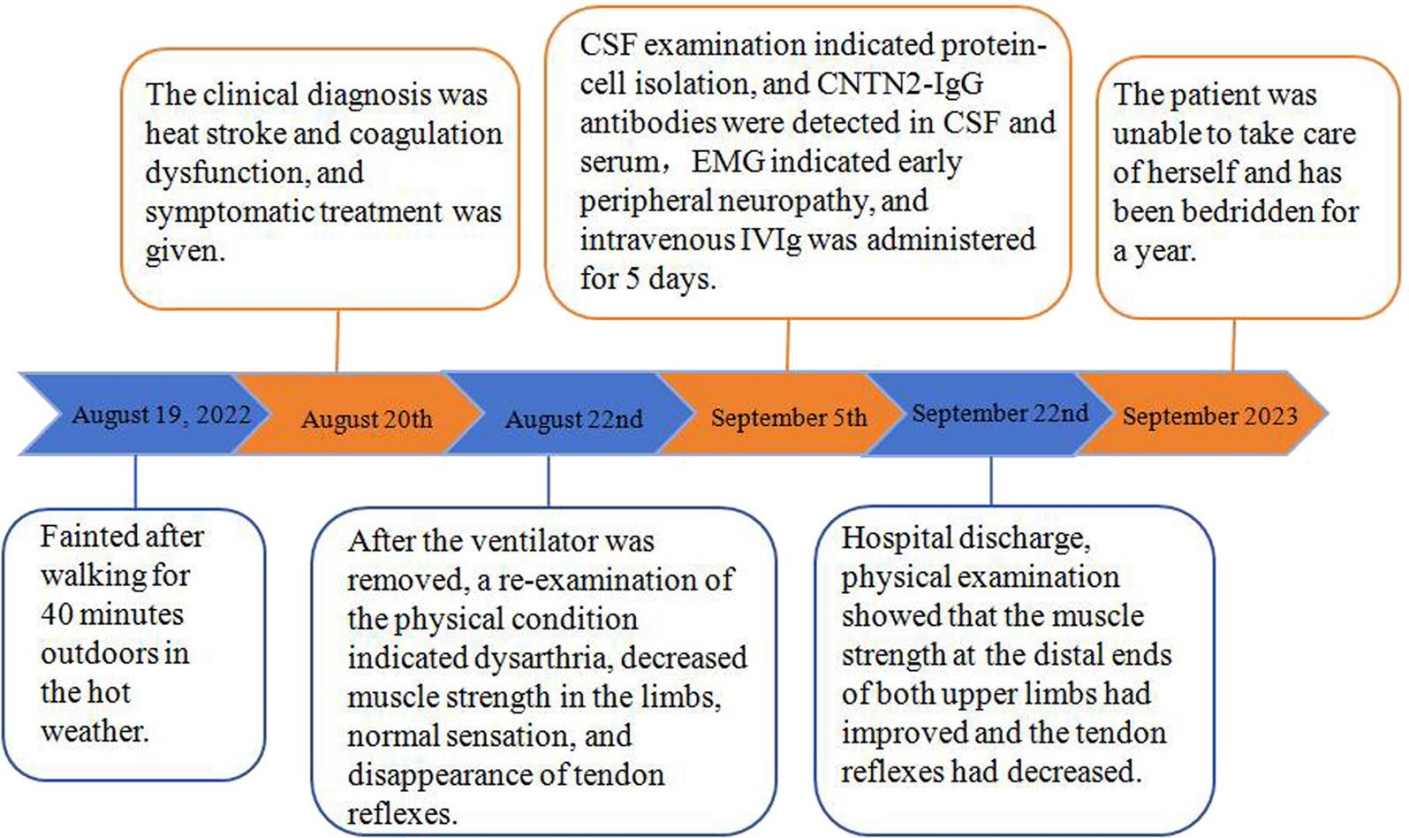
The clinical timeline of the patient

At the time of discharge, the patient was delirious and shaken her head while receiving oxygen through the tracheostomy cannula. Muscle strength in both upper limbs was graded as 2, whereas that in both lower limbs was graded as 1. During a follow-up conducted via telephone 1 year later, in September 2023, the patient remained unable to care for herself and required long-term bed rest (Fig. 2).

## Discussion

There are currently no documented cases globally of peripheral neuropathy linked to positive anti-contactin-2 (CNTN2) antibodies. This study aims to elucidate the clinical diagnostic and therapeutic characteristics by synthesizing existing literature.

Myelinated axons within peripheral nerves are enveloped by myelin sheaths produced by Schwann cells. The gap between adjacent myelin sheaths is termed the node of Ranvier, which is bordered by microvilli from Schwann cells, while the paranodal junctions are surrounded by cytoplasm-filled myelin sheaths [1]. The node is densely populated with neurofascin 186 (NF186), neural cell adhesion molecules (NCAM), voltage-gated sodium channels (Nav), and neurofascin, all of which contribute to the structural integrity of the node. Within the myelin sheath, neurofascin 155 (NF155), contactin-associated protein 1 (Caspr1), and contactin-1 (CNTN1) form a complex that securely anchors the myelin sheaths to the axolemma. This compartmentalized architecture ensures that the ion channel proteins at the node do not interfere with the voltage-gated potassium channels located in the juxtaparanode (KV1.1–KV1.2) [2].

In the juxtaparanode, contactin-2 (CNTN2), contactin-associated protein 2 (Caspr2), postsynaptic density proteins 93/95 (PSD93/95), and the actin-spectrin cytoskeleton (primarily via protein 4.1B) form a complex that anchors the voltage-gated potassium channels to the paranodal cytoskeleton [3, 4].

CNTN2 antibodies play a significant role in multiple sclerosis, as studies of nerve tissue from affected patients and mouse models have demonstrated severe damage to the juxtaparanode [5]. CNTN2 was originally identified during axonal growth and was designated transient axonal glycoprotein-1 (Tag-1) because of its transient expression. The function of CNTN2 is not fully understood; studies suggest that CNTN2 acts as a glycosylphosphatidylinositol (GPI) linker protein and an axon adhesion molecule of the immunoglobulin superfamily, connecting to axons or myelin sheaths through GPI anchors [5]. In the juxtaparanode, CNTN2 and Caspr2, which are located in axons, interconnect with CNTN2 on myelin sheaths. Caspr2 forms Caspr2/Kv complexes with the voltage-gated KV1.1 and KV1.2 channels. The transmembrane receptors disintegrin and metalloproteinase domain-containing protein 22 (Adam22) and disintegrin and metalloproteinase domain-containing protein 23 (Adam23) interact with leucine-rich glioma-inactivated protein 4 (LGI4) to recruit PSD-93/95, whereas PSD-95 associates with KV1.1, which is mediated by Caspr2. In the cytoplasm, Caspr2 binds to protein 4.1B, αII spectrin, and β2 spectrin. Therefore, a complex comprising CNTN2, Caspr2, and PSD93/95 anchors the KV channel to a cytoskeleton composed of 4.1B, αII spectrin, and βII spectrin, thereby helping to maintain the internodal resting potential. In the absence of CNTN2, Caspr2 fails to accumulate at the juxtaparanode, resulting in a deficiency of KV channels [5–9].

CNTN2 (also referred to as Tag-1) is expressed on developing axons and plays a crucial role in the migration of motor neurons, the formation of axon bundles, and the guidance of axonal extension and localization [10, 11]. CNTN2 regulates axonal responses to stretch signals by modulating the endocytosis of its receptors [12]. Numerous studies on cellular and molecular biology have demonstrated that the proliferation and migration of Schwann cells significantly contribute to axonal growth and functional recovery following peripheral nerve injury. CNTN2 serves as a regulatory target for Schwann cells, promoting nerve regeneration, whereas miR-34a and miR-3075 inhibit Schwann cell proliferation, migration, and axonal growth by negatively regulating CNTN2 expression [13, 14].

Research conducted by Zoupi indicated that CNTN2 affects the morphology of oligodendrocytes, myelination, and axonal transmission properties of the corpus callosum white matter tract. This study revealed that when electrical stimulation was applied to mutant mice lacking the CNTN2 gene, both the frequency and amplitude of the compound action potential significantly decreased. This phenomenon may reflect corresponding structural changes or the number of axons [15]. Numerous studies have demonstrated that CNTN2 is transiently expressed on the bodies of migrating motor neurons and localizes to distal motor axons during neurite extension in the peripheral nervous system during embryonic development. CNTN2 is involved in regulating axon growth, localization, and fasciculation, and it can control motor neuron localization by mediating the Sema6A-Nrp2 or Netrin5-DCC signalling pathways [10, 11]. On the basis of the patient's course and symptoms, we hypothesized that the following pathophysiological mechanisms contributed to the reduction in CNTN2 and the decrease in the levels of complexes formed with Caspr2, PSD93/95, and 4.1B. This reduction likely led to a decreased density of KV channels recruited by these complexes, resulting in an inability of the cells to repolarize and consequently causing axonal damage. We administered gamma globulin to the patient to eliminate CNTN2 antibodies, facilitating the production of new CNTN2, which reanchored the KV channel to the juxtaparanode and improved the patient's symptoms. The pathogenesis of CNTN2 antibodies remains unclear, necessitating further in-depth studies.

The disease presented with an acute onset, marked by the presence of CNTN2 antibodies in both cerebrospinal fluid and serum, with higher titres observed in the serum. The patient experienced an acute onset with an incubation period of merely 3 days and had been in good health prior to this. Two weeks post-onset, routine cerebrospinal fluid analyses and electromyography revealed protein-cell dissociation and axonal damage, indicative of Guillain-Barré Syndrome (GBS). Given the severity of the patient's condition, a chest CT scan demonstrated minor pulmonary inflammation. As a precautionary measure, intravenous immunoglobulin (IVIg) therapy was administered; however, the patient's symptoms did not improve. Electrophysiological evaluation confirmed axonal peripheral neuropathy, suggesting that CNTN2 antibodies may induce autoimmune nodopathy, predominantly affecting motor nerves. Peripheral neuropathy associated with CNTN2 and CNTN1 progresses rapidly, with evidence of axonal involvement detectable early in the disease course. Some studies suggest that 60% of patients with CNTN1-associated peripheral neuropathy also experience nephropathy [16, 17]. CNTN2 is associated with multiple sclerosis, as well as autosomal recessive cortical myoclonic tremor and epilepsy [5, 18]. Research indicates that AN is primarily mediated by IgG4, the IgG 4 may be mostly by CD20 positive B cells, previous studies on the structure and function of IgG4 and its role in autoimmune neurological disorders have shown that IgG4 antibodies exhibit noninflammatory properties. They are unable to cross-link immune complexes to degrade antigens via activation of cellular or complement-mediated immune pathways. Instead, their pathogenicity mainly involves directly blocking protein–protein interactions, thereby affecting antigen structure and function. Consequently, as IgG4 lacks typical proinflammatory antibody functions, glucocorticoids and IVIg are often less effective [19, 20]. The main mechanisms of IVIg include the following: idiotypic antibodies within IVIg can bind to and neutralize pathogenic autoantibodies, preventing their interaction with autoantigens; IVIg accelerates IgG catabolism by saturating protective FcRn receptors, which are highly expressed on vascular endothelial cells; it inhibits complement binding and prevents membranolytic attack complex (MAC) formation; it may suppress CD8-mediated cytotoxicity and T-cell apoptotic molecules, further inhibiting activated T-cells. Moreover, as the blood–nerve barrier is impaired at nerve roots and distal terminals, IVIg can freely enter these areas and may exert additional local effects on the respective tissues [21], and so on. One study reported that autoantibodies in all NF155 + AN patients were predominantly of the IgG4 subclass. Among these patients, only 13.1% responded well to IVIg, whereas 77.3% responded well to rituximab (RTX). Antibody titers decreased by approximately 66.7% as early as 3 months after RTX initiation [22]. IgG4 is secreted exclusively by IL-10 + regulatory B-cells [23]. As an immunosuppressant that depletes B cells [24], RTX can effectively inhibit IgG4 production, which explains its favorable effect in IgG4-mediated autoimmune neurological diseases. However, effective treatments for CNTN2 + AN remain insufficiently studied. Additionally, the patient's admission chest CT indicated mild pulmonary inflammation, prompting the administration of intravenous immunoglobulin (IVIg) as a safety treatment, delaying the timely administration of rituximab (RTX). This decision underscores the importance of understanding pathophysiology and conducting clinical research to inform treatment strategies. Once IgG4-related autoimmune nodopathy (AN) is diagnosed, RTX should be administered promptly, following the exclusion of contraindications, to alleviate symptoms.

## Conclusion

This report is likely the first to document autoimmune nodopathy associated with contactin-2 (CNTN2) antibodies. We provide a comprehensive summary of the clinical symptoms, electrophysiological examinations, diagnoses, treatments, and follow-up outcomes. Furthermore, we explored the function of CNTN2 and compared it with CNTN1-induced autoimmune nodopathy (see Table 2). This study has several limitations, including the lack of investigation into the relationship between heat stroke and CNTN2 antibody-induced autoimmune nodopathy, the absence of reexamination of CNTN2 antibodies through lumbar puncture and blood sampling, and the omission of imaging and pathology assessments. CNTN2 antibodies were detected in the cerebrospinal fluid (CSF) of this patient, but the reason for their presence remains unclear.

**Table 2:** Comparison of contactin-2 antibody positive and contactin-1 antibody positive peripheral neuropathy

Type	CNTN1	CNTN2
Onset of illness	Acute	Acute
Common symptoms	Limb motor function defect, sensory ataxia, tremor, neuralgia	Limb motor deficits, dysarthria, respiratory failure (central involvement of anti-CNTN2 antibodies)
Affected nerves	Mainly motor nerve	Mainly motor nerve
Electrophysiology	Early axonal involvement was detected and motor and sensory nerve conduction velocities were reduced	In the early stage, axonal involvement was detected, and motor nerve conduction velocity and amplitude decreased
Complications	Membranous nephropathy	–
Treatment	Cyclosporine; rituximab	Immunoglobulin
Imaging	Enlarged cervical and lumbar nerve roots	–
Pathological features	Severe axonal degeneration, Langerhans node prolongation, subperineal edema, no demyelination and remyelination	–

## Data Availability

The datasets during the study available from the corresponding author on reasonable request.
